# Transformational Leadership, Ethical Leadership, and Participative Leadership in Predicting Counterproductive Work Behaviors: Evidence From Financial Technology Firms

**DOI:** 10.3389/fpsyg.2021.658727

**Published:** 2021-08-04

**Authors:** Stanley Y. B. Huang, Ming-Way Li, Tai-Wei Chang

**Affiliations:** ^1^Master Program of Financial Technology, School of Financial Technology, Ming Chuan University, Taipei City, Taiwan; ^2^Department of Marketing and Logistics Management, College of Business Management, Chihlee University of Technology, New Taipei City, Taiwan; ^3^Graduate School of Resources Management and Decision Science, National Defense University, Taoyuan, Taiwan

**Keywords:** counterproductive work behaviors, employee engagement, participative leadership, ethical leadership, transformational leadership

## Abstract

Counterproductive work behaviors are a crucial issue for practice and academic because it influences employees’ job performance and career development. The present research conceptualizes Kahn’s employee engagement theory and employs transformational leadership, ethical leadership, and participative leadership as its antecedents to predict counterproductive work behaviors through a latent growth model. The present research collected empirical data of 505 employees of fintech businesses in Great China at three waves over 6 months. The findings revealed that as employees perceived higher transformational leadership, ethical leadership, and participative leadership at the first time point, they may demonstrate more positive growths in employee engagement development behavior, which in turn, caused more negative growths in counterproductive work behaviors. The present research stresses a dynamic model of the three leaderships that can alleviate counterproductive work behaviors through the mediating role of employee engagement over time.

## Introduction

Previous empirical studies on the antecedents that can mitigate negative work behaviors in Greater China lack sufficient research (Lee and Huang, 2019; Wen et al., 2020; Zeng et al., 2020; Lan et al., 2021), thereby indicating the importance of exploring key antecedents of counterproductive work behaviors (CWBs) (Baloch et al., 2017; Chen et al., 2017; Tziner et al., 2020; Yan et al., 2020). Moreover, CWBs are an important concept, because CWBs may cause lost productivity and withdrawal. In particular, the employees of fintech firms in Greater China have high levels of pressure in recent decades, because Greater China has become one of the most onerous countries for fintech business (Dai and Taube, 2019). For example, the world’s top one, top three, and top six fintech firms are, respectively, Ant Financial, JD Digits, and Du Xiaoman Financial in Greater China (KPMG, 2019). Therefore, it is crucial to understand which organizational management mechanisms (e.g., leadership) can effectively deal with the CWBs. CWBs denote an employee behavior that harms colleagues or company to respond to strain and give vent to an emotion of disagreement (Hollinger and Clark, 1983). Previous studies have almost examined the antecedents of CWBs using personal variables (e.g., Zhou et al., 2014; Bowling and Lyons, 2015; Fida et al., 2018), so it lacks a complete complementary theory to predict CWBs (Lasson and Bass, 1997). Therefore, the present research borrows from Kahn’s (1990) engagement theory as a theoretical basis to predict CWBs. Employee engagement (EE) denotes that an employee putting all resources into self-concept toward a job role to achieve a high level of performance (Kahn, 1990). The EE is also gradually received the attention of Greater China scholars (Lan et al., 2020; Liu et al., 2020; Lyu, 2020). In addition, Kahn’s (1990) theory has detected three psychological antecedents that can drive an individual to show EE, and the present research suggests transformational leadership (TL), ethical leadership (EL), and participative leadership (PL) as Kahn’s (1990) three psychological antecedents. In particular, the TL, EL, and PL are three leadership styles in different domains. For example, the three leadership styles (Bass, 1985; Brown et al., 2005; Somech, 2006) are involved employees’ different implementation of TL (e.g., adaptability, new ideas, and job performance), EL (e.g., honesty, fairness, trust, and consideration), and PL (openness, authorization, and autonomy). Although EL has been found it is correlated to the idealized consideration dimension of TL (Brown et al., 2005), the correlation coefficient is trivial (0.19), thereby indicating a significant difference between the TL and EL.

In sum, the present research proposes the theoretical model that higher levels of TL, EL, and PL at the first time point will cause more positive growths in the EE, and more positive growths in the EE will cause more negative growths in CWBs over time. The present research employs the latent growth curve modeling (LGCM) and longitudinal data at three-time points in 6 months to verify the theoretical model of the present research based on Kahn’s (1990) theory, which can also complement past knowledge gaps.

## Theory Development and Hypotheses

### Kahn’s Theory

EE denotes “simultaneous employment and expression of a person’s preferred self in task behaviors that promote connections to work, others, personal presence (physical, cognitive, and emotional) and full performances” (Kahn, 1990, p. 700). That is to say, EE denotes that an employee’s emotional, cognitive, and physical resources into a job to achieve high levels of job performance (Basinska and Dåderman, 2019; Holmberg et al., 2020).

### Antecedents of EE

In Kahn’s (1990) survey, three psychological states were found to influence an individual’s decision whether to perform EE, containing availability, safety, and meaningfulness. The meaningfulness denotes whether an employee’s self-worth is consistent with the company. Safety denotes whether an employee’s working environment is trustworthy and safe. The availability denotes whether an employee has confidence enough to perform the work. The present research employs TL, EL, and PL as drivers of meaningfulness, safety, and availability.

The TL of supervisors can increase their followers’ psychological meaningfulness of Kahn (1990). TL denotes that a leader employs four dimensions (intellectual stimulation, individual consideration, idealized influence, and inspirational motivation) to guide followers to achieve a high level of thinking (Bass, 1985) and has been widely applied in organizational research (e.g., Wang et al., 2018; Tian et al., 2020; Zhang et al., 2021). Based on previous research, TL can transmit organizational values to their followers and can also transform followers’ self-worth to meet companies’ values (Shamir et al., 1993; Bono and Judge, 2003), thereby indicating the relationship between TL and EE. That is to say, when employees’ self-worth is consistent with the companies’ values, they will perceive high levels of meaningfulness, which will influence these employees to show higher levels of EE. A past study also supported this argument (Chen and Huang, 2016). Therefore:

*Hypothesis 1: A follower who perceived higher levels of TL at the first time point will lead to more positive growths in EE over time.*

The EL of supervisors can increase their followers’ psychological safety that Kahn (1990) proposes as an antecedent of engagement. Previous researchers have proposed different definitions of EL (Resick et al., 2006; Kalshoven et al., 2011; Eisenbeiss and Giessber, 2012), and the present research employs Brown et al.‘ (2005) definition that described EL as a “demonstration of normatively appropriate conduct through personal actions and interpersonal relationships, and the promotion of such conduct to followers through two-way communication, reinforcement, and decision-making” (Brown et al., 2005, p. 120) because of its universality. EL denotes the leaders’ capability in attracting their followers to meet ethical standards (Ruiz et al., 2011; Steinmann et al., 2016; Metwally et al., 2019; Fu et al., 2020; Manara et al., 2020) through social learning theory (Bandura, 1986). That is to say, these behaviors of followers can meet ethical standards because EL of supervisors attracts these followers to learn and imitate ethical leaders’ favorable behaviors, such as integrity and morality, to form the ethical identity.

Previous researchers have found that employees may feel their work environments are safe and supported because of EL (Brown et al., 2005). EL also can reduce followers’ anxiety toward the uncertainty of work environments because EL can shape trustworthy, open, and honest atmosphere (Treviño et al., 2003). Therefore, EL can make employees feel safe in their work environments, thereby indicating the relationship between EL and EE. Indeed, Previous studies also indicated that EL can arouse positive workplace behaviors and these positive workplace behaviors are like EE (Ruiz et al., 2011; Sharif and Scandura, 2014; Metwally et al., 2019). Therefore:

*Hypothesis 2: A follower who perceived higher levels of EL at the first time point will lead to more positive growths in EE over time.*

The PL of supervisors can increase their followers’ psychological availability that Kahn (1990) proposes as an antecedent of engagement. PL denotes that a leader gives followers a high degree of authority to let them decide how to perform work tasks (Somech, 2006), which must strengthen these subordinates’ assessment of self-confidence. Indeed, these subordinates must perceive high levels of self-confidence because they perceive high participative leadership from their supervisor, and, in turn, they own more available resources to invest in their job roles to show high levels of EE. Therefore:

*Hypothesis 3: A follower who perceived higher levels of PL at the first time point will lead to more positive growths in EE over time.*

### EE and CWBs

Previous researchers (e.g., Colbert et al., 2004; Dalal, 2005) found that CWBs and their antecedents can be explained by social exchange theory and norm reciprocity theory (Gouldner, 1960; Levinson, 1965). That is to say, employees may use good or bad behaviors to respond to their companies because these employees receive good or bad treatments from their companies based on the social exchange theory and norm reciprocity theory. Indeed, if employees receive bad treatment from their companies, they may retaliate by the CWBs to give vent to an emotion of disagreement (Bushman et al., 2001).

Previous researchers have confirmed multiple job-related attitudes can cause CWBs (Shepard and Durston, 1988; Huang et al., 2017), and these job-related attitudes overlap with EE. Indeed, EE includes physical involvement, emotional connection, and cognitive connection to a job, and must contain most job-related attitudes (Macey and Schneider, 2008). The second possible reason that EE can be an antecedent of CWBs is that positive emotions must negatively influence CWBs (Fox et al., 2001) and engaged employees must have high levels of enthusiasm to infect their colleagues (Bakker, 2008), thereby indicating the relationship between EE and CWBs. Therefore:

*Hypothesis 4:* The more positive growths in EE will lead to more negative growths in CWBs over time.

### The Mediating Effect of Employee Engagement

Previous researchers have proposed that TL can reduce the likelihood of CWBs (Rajnandini et al., 1999; Kessler et al., 2013; Mekpor and Dartey-Baah, 2017). In addition, EL can guide followers to meet the moral standard, so they should reduce the likelihood of CWBs, which is also supported by a previous study (Bakker et al., 2004). In the same vein, past studies also argued that PL has a similar effect on CWBs (Li et al., 2017, 2019). Therefore:

*Hypothesis 5: The EE plays a* mediating role between TL, EL, and PL to CWBs.

## Methodology

**FIGURE 1:**
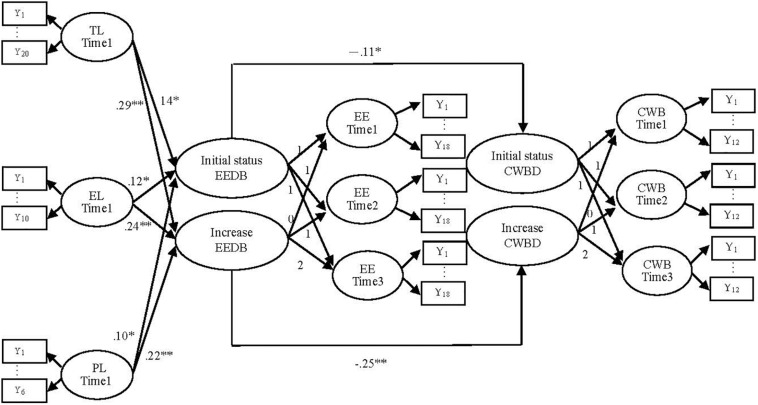
The latent growth model of this study. TL, Transformational leadership; EL, Ethical Leadership; PL, Participative Leadership; EE, Employee Engagement; CWB, Counterproductive Work Behaviors; EED, Employee Engagement development; CWBD, Counterproductive Work Behaviors development; Y_*n*_, Measurement items. ^∗^*p* < 0.05; ^∗∗^*p* < 0.01.

The proposed model of the present research (Figure 1) is from TL, EL, and PL to CWBs based on Kahn’s (1990) theory.

### Sample Procedures

The present research obtained longitudinal data through three-time points from the employees of fintech businesses in Greater China. The present research collected data at an individual level because the present research conceptualizes Kahn’s (1990) work by the latent growth model with longitudinal data rather than to explore the cross-level framework. In particular, there is no strong evidence to support that Kahn’s (1990) work can be conceptualized as a multilevel model. The present research contacted to collect a list of financial technology firms and requested these supervisors to invite their followers. Before filling out the questionnaire, the present research informed them about the sampling process. Each respondent would receive a coupon worth about US$4 as a reward if they finished the sampling process. When the present research received the first time point data for the assessments about employees’ TL, EL, PL, and EE, and their supervisors’ assessments about these employees’ CWBs at the first time point, the present research investigated these employees’ assessment about the perception of EE and their supervisors’ assessments about theses employees’ CWBs at 3 months later (the second time point). The present research collected the third time point data of the employee assessments about EE and these supervisors’ assessments about CWBs 6 months later. The present research got samples of 505 employees and their supervisors. That is to say, there is no nested framework (Raudenbush and Bryk, 2002) in the present research, and an individual-level statistical technique (e.g., latent growth model) rather than a cross-level statistical technique (e.g., hierarchical linear modeling) should be employed to test the samples. Based on the valid samples, 59% are male and the average age is 28 years. In addition, 90% have a college education. The sample design with multiple sources and time can reduce common method bias (Podsakoff et al., 2003, 2006, 2016).

### Measures

The present research adopted a 7-point Likert to assess the items of TL, EL, PL, EE, and CWBs and adopted backward translation to confirm the translation quality (Brislin, 1986).

TL was measured by Bass and Avolio’s (1995) scale with twenty-item. EL was measured by Brown et al.’s (2005) scale with 10-item. PL was measured by Arnold et al. (2000). scale with six-item. EE was measured by Rich et al. (2010) scale. CWBs was measured by Dalal et al. (2009) scale.

### Data Analysis

#### LGCM

The LGCM is used to analyze repeat measurement of variables and how changes in these variables can lead to changes in other variables (Duncan et al., 2006). That is to say, the good point of LGM is that it can effectively analyze the relationship changes between variables. For example, the present research investigated the three-time point data, and LGCM can capture the changes in TL, EL, PL, EE, and CWBs, and analyze the causal relationship between these variables. The parameter setting of factor loadings for these variables can refer to Duncan et al. (2006).

The factor loadings of these variables in the present research are fixed in 1 because it can evaluate the intercept of the variables through the three-time points equally. The factor loadings of these variables in the present research are fixed to 0, 1, and 2 because they can measure positive increases in these variables (Duncan et al., 2006). In addition, the first loading is fixed at 0, because it can reflect the average value of the first time point. For example, based on Figure 1, the initial status EEDB is related to EE at the three-time points with the loadings fixed in 1 because initial status EEDB means an average value of the three-time points. Besides, increase EEDB is related to EE of the three-time points with the loadings fixed at 0, 1, and 2 because increase EEDB means positive changes over EE of the three-time points. To detect the relationship between increase EEDB and increase CWBD, the path from increase EEDB to increase CWBD is added to estimate the effect (β = −0.25, *p* < 0.01). Based on Hair et al. (2016), the present research employed LGCM with longitudinal data to analyze the theoretical model because most past studies were cross-sectional surveys.

### Confirmatory Factor Analysis

The present research adopted the confirmatory factor analysis to analyze the composite reliability (CR), the average variance extracted (AVE), and the model fit. The AVE of TL (first time point), EL (first time point), PL (first time point), EE (first time point to third time point), CWB (first time point to third time point) are, respectively, 0.63, 0.61, 0.58, 0.63, 0.69, 0.61, 0.55, 0.59, and 68. In the same vein, these variable in different time point are, respectively, 0.69, 0.71, 0.75, 0.7, 0.72, 0.69, 0.62, 0.61, and 72. The model fit indexes (RMR, RMSEA, GFI, CFI, NFI) of the theoretical model in this study are, respectively, 0.5, 0.4, 0.93, 0.91, and 0.92. RMR and RMSEA are the appropriateness of the theoretical model and the real data (Hu and Bentler, 1999), and it is similar to the error in the statistical regression technique. GFI is employed to measure how well a specified model reproduces the covariance matrix among the indicator variables (Hu and Bentler, 1999), and it is similar to the R^2^ (explain variation) in the statistical regression technique. CFI is an improvement index, and it is defined as the degree of improvement between the theoretical model of this study and the independent model (Hu and Bentler, 1999). NFI is also an improvement index, and it is defined as the degree of improvement between the best model and the worst model (Hu and Bentler, 1999). The present research chose RMSEA, GFI, CFI, and NFI to evaluate the fit between empirical data and the theoretical model in this study because these fitness indices have been suggested by past studies to confirm an explanatory power of the model (Fornell and Larcker, 1981; Hu and Bentler, 1999; Kenny and McCoach, 2003). In sum, the AVE, CR, model fit indexed are all above the threshold suggested by previous studies (Fornell and Larcker, 1981; Hu and Bentler, 1999; Kenny and McCoach, 2003). Table 1 provides the correlation of all variables. Besides, the rwg(j) (James et al., 1984) of TL, EL, PL, EE, and CWBs are all less than 37, which indicates that there is no cross-level framework in the present research.

**TABLE 1:** Means, standard deviations, and correlation (*N* = 1,105).

	M	SD	TL	EL	PL	EE	CWB
TL	3.91	0.89					
EL	3.52	0.81	0.19				
PL	3.12	0.91	0.17	0.21			
EE	3.79	0.88	0.39	0.34	0.31		
CWB	3.43	0.91	0.23	0.20	0.19	0.44	

**TL, Transformational Leadership; EL, Ethical Leadership; PL, Participative Leadership; EE, Employee Embeddedness; CWB, Counterproductive Work Behaviors.**

### The Results of the Analysis

The TL, EL, and PL at the first time point were related to the increase and initial status of EE (please see Figure 1). The TL (β = 0.29, *p* < 0.01), EL (β = 0.24, *p* < 0.01), and PL (β = 0.22, *p* < 0.01) at the first time point significantly caused more growths in EE over time. Hypothesis 1, 2, and 3 are supported, and these hypotheses assume that followers who perceived higher levels of TL, EL, PL at the first time point would show more growths in EE over time. That is to say, these followers have already developed EE when they perceived TL, EL, and PL at the first time point.

The increase and initial status of EE were related to the increase and initial status of CWBs (please see Figure 1). The more growths in EE would significantly lead to more growths in CWBs over time (β = −0.25, *p* < 0.01). Hypothesis 4 is supported.

Baron and Kenny’s (1986) three-step method was used to test the mediating effect of EE in the proposed present research. Although subsequent research has developed a new test method, the present research believes that the method of Baron and Kenny is still robust. Based on the three-stage analysis suggested by Baron and Kenny (1986), and the analysis results showed the coefficients from TL, EL, and PL to CWBs were not significant when the EE was added into the model, thereby indicating a mediating effect of EE. In addition, the Chi-Squared difference test was also used to test the mediating effect of EE, and the results also supported the mediating role of EE. Finally, the present research employed the Sobel test (Sobel, 1982) to analyze the effect, and the analysis results showed significant indirect effects. Therefore, hypothesis 5 is supported.

## Discussion

### Academic Contribution

First, the contribution of the present research is to demonstrate the growths in EE and CWBs using LGCM to advance the literature. In particular, Kahn’s engagement theory is used to support the theoretical model of the present study. Based on the empirical results, the CWBs are explained by the three leadership styles and EE well.

Second, in Kahn’s (1990) study, he described “engagement in terms of dynamic moments,” ebbs and flows, and “calibrations of self-in-role” (1990, p. 694). That is to say, EE may fluctuate over time, and the present research uses LGCM with longitudinal data over 6 months to respond to Kahn’s (1990) call.

Finally, intervention strategies for CWBs focus primarily on personal variables (e.g., individual characteristics) in past studies (e.g., Zhou et al., 2014; Bowling and Lyons, 2015; Fida et al., 2018). These personal variables can alleviate CWBs, but they only can help companies to recruit new employees with low risk characteristics. However, organizational interventions strategies (e.g., leadership) for CWBs may be more effective to deal with CWBs of employees.

### Practical Contribution

According to the analysis results of the present study, human resource managers should not invest most resources in recruiting new employees who fit job requirements, but should first invest in the educational training of leadership for supervisors to increase their TL, EL, and PL. Indeed, retaining an old employee is more valuable than recruiting a new employee, because the old employee with high levels of CWBs may leave the company.

Another key variable that can reduce CWBs is EE, so human resource managers should also think about how to invest resources in programs that can increase EE. For example, establishing a good working environment and a fair system can make employees feel safe and further increase their EE. In addition, self-confidence courses can be integrated into employee education training to increase their availability and thereby increase their EE. Finally, supervisors should always communicate with employees the company’s strategy and vision to increase the meaningfulness of their work, thereby increasing EE.

### Limitations

First, the present study employs a narrow concept to represent the CWBs, but other negative behaviors are not included in CWBs. Second, the antecedent of EE in the present study are TL, EL, and PL, but there should be another important antecedents in different contexts. T Finally, the Great China sample in the present study also shows its limitations. The fourth limitation of the present research is that although the present research employs a multi-source design (employees and supervisors) to obtain data from the self-rating questionnaire, future investigations should use a better method to handle common method variance. Finally, the present research surveyed longitudinal data in 6 months to analyze the theoretical model, but the hypotheses of the present research should have more longitudinal data to confirm the causal inference.

### Further Research

As the above discussion of limitation, the present research proposes three suggestions for future study. First, further research should examine the proposed model of the present study in different countries to increase the generalization. For example, the culture of effectiveness (Metwally et al., 2019) may influence the theoretical model in the present research (e.g., ethical leadership, and its outcomes) and the present research encourages other researchers to test the effect of cultures of effectiveness to increases the generalization of the present research Second, further research should explore different antecedent of EE in different contexts to advance the literature of EE because different antecedents should be considered in different contexts. Finally, the common method variance cannot be eliminated because of the self-rating questionnaire and the present research suggests a new measurement method should be employed in handling this problem, such as the eye-tracking technique (Holmqvist et al., 2011).

## Data Availability Statement

The original contributions presented in the study are included in the article/supplementary material, further inquiries can be directed to the corresponding author/s.

## Author Contributions

SH was mainly responsible for the content writing of the manuscript. M-WL assisted in sampling and manuscript revision. T-WC assisted in the literature collection. All authors contributed to the article and approved the submitted version.

## Conflict of Interest

The authors declare that the research was conducted in the absence of any commercial or financial relationships that could be construed as a potential conflict of interest. The reviewer SL declared a shared affiliation with the authors to the handling editor at the time of review.

## Publisher’s Note

All claims expressed in this article are solely those of the authors and do not necessarily represent those of their affiliated organizations, or those of the publisher, the editors and the reviewers. Any product that may be evaluated in this article, or claim that may be made by its manufacturer, is not guaranteed or endorsed by the publisher.
